# Supplementary figures and images for: The prion protein is not required for peripheral nerve de- and remyelination after crush injury
Source: PLoS One. 2021 Jan 22;16(1):e0245944. doi: 10.1371/journal.pone.0245944 (PMC7822300; doi:10.1371/journal.pone.0245944)

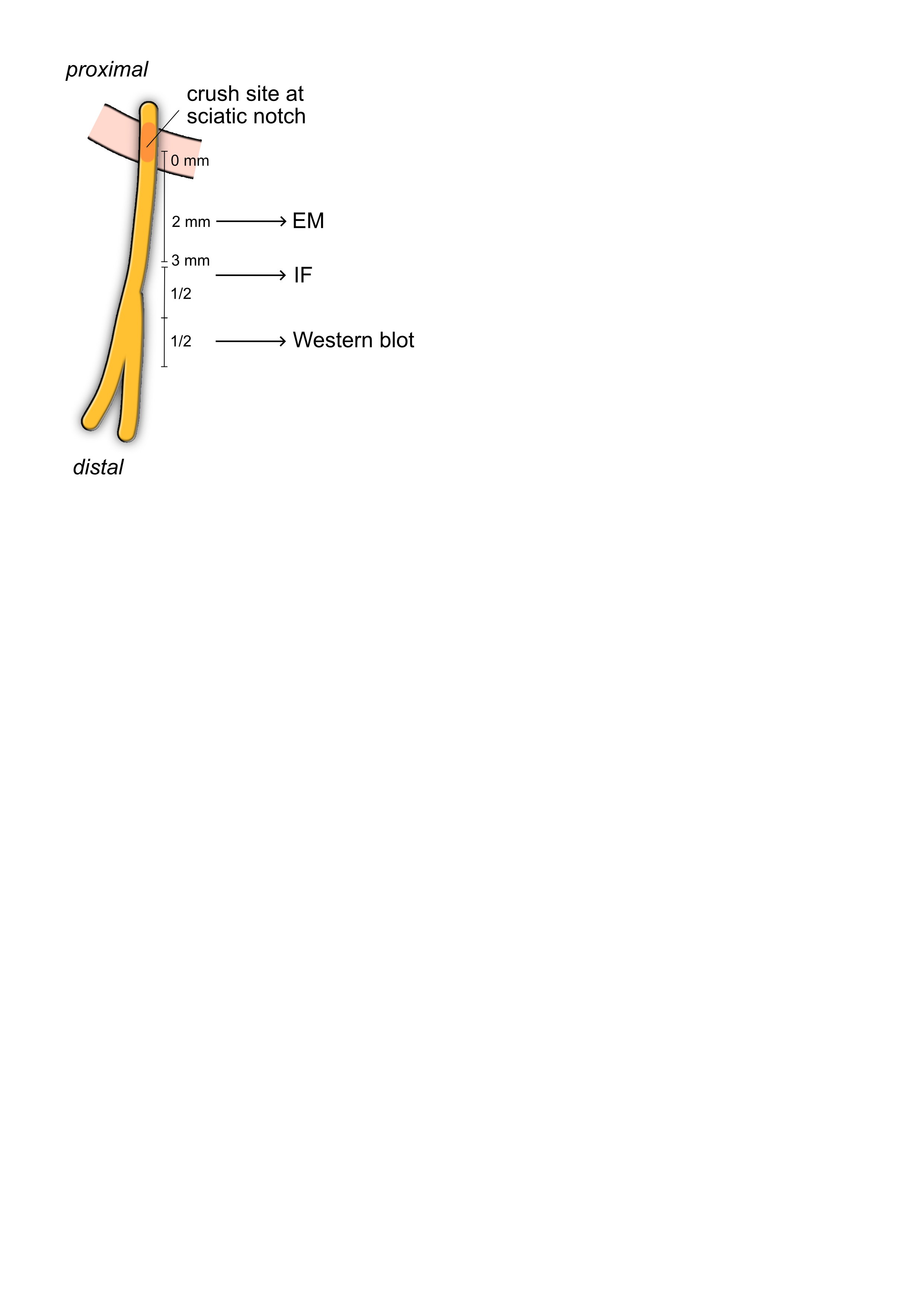

Supplement: S1 Fig — The sciatic nerve was crushed using a forceps at the sciatic notch. For harvesting, the nerve was cut at 3 mm distal to the crush side. The proximal segment was embedded for electron microscopy (EM), the distal segment was used for immunofluorescence (IF) and western blotting. (TIFF) [file pone.0245944.s001.tiff]

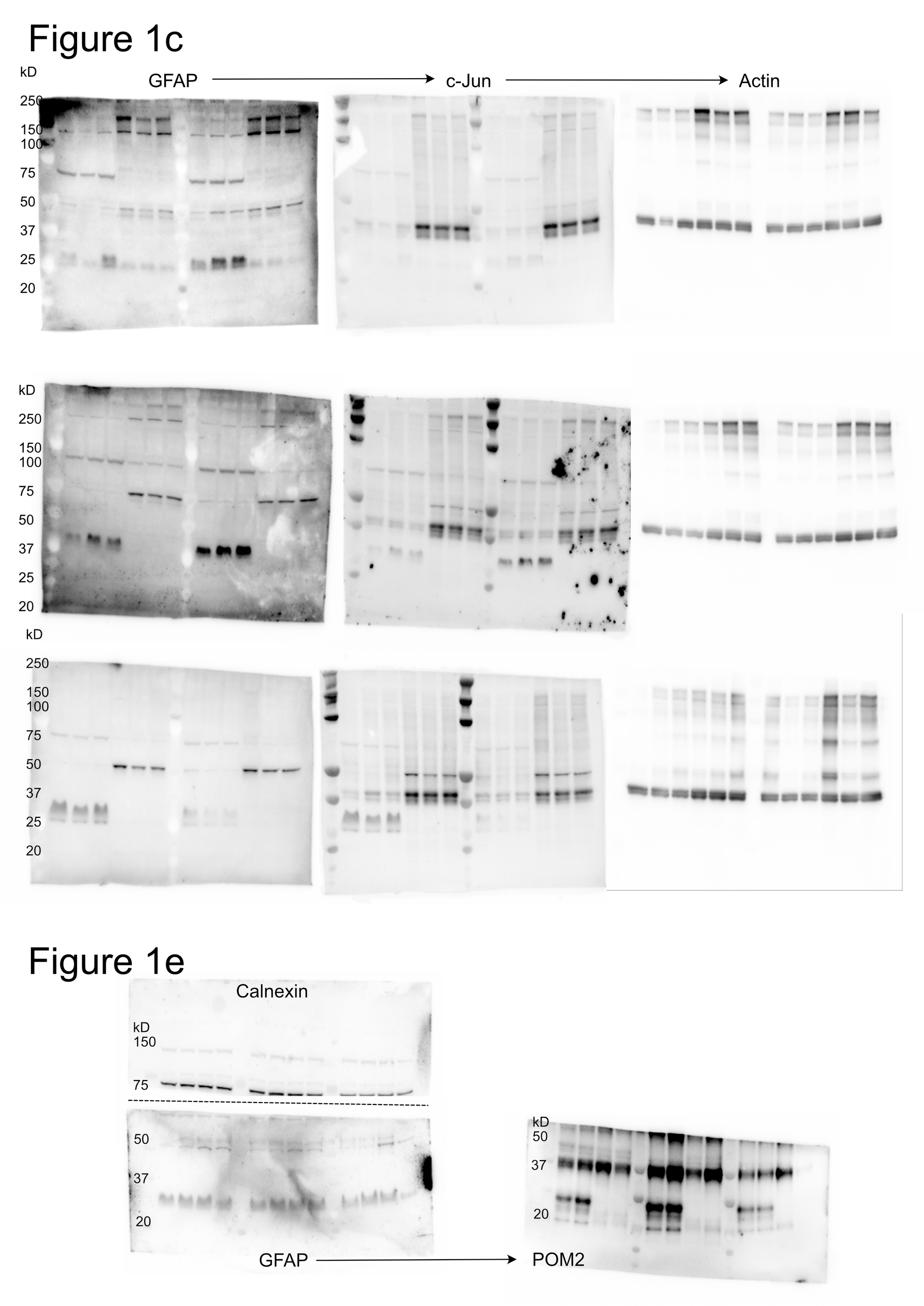

Supplement: S2 Fig — The uncropped blots have been inverted using the Affinity Photo software. Molecular weight markers used are indicated in kilo Dalton (kD). The arrows indicate the order in which the blots were incubated with primary antibodies. Stripping was performed before each incubation. The dashed line in blot Fig 1e indicates that the membrane was cut, and the upper and lower half were incubated with different antibodies. (TIF) [file pone.0245944.s002.tif]

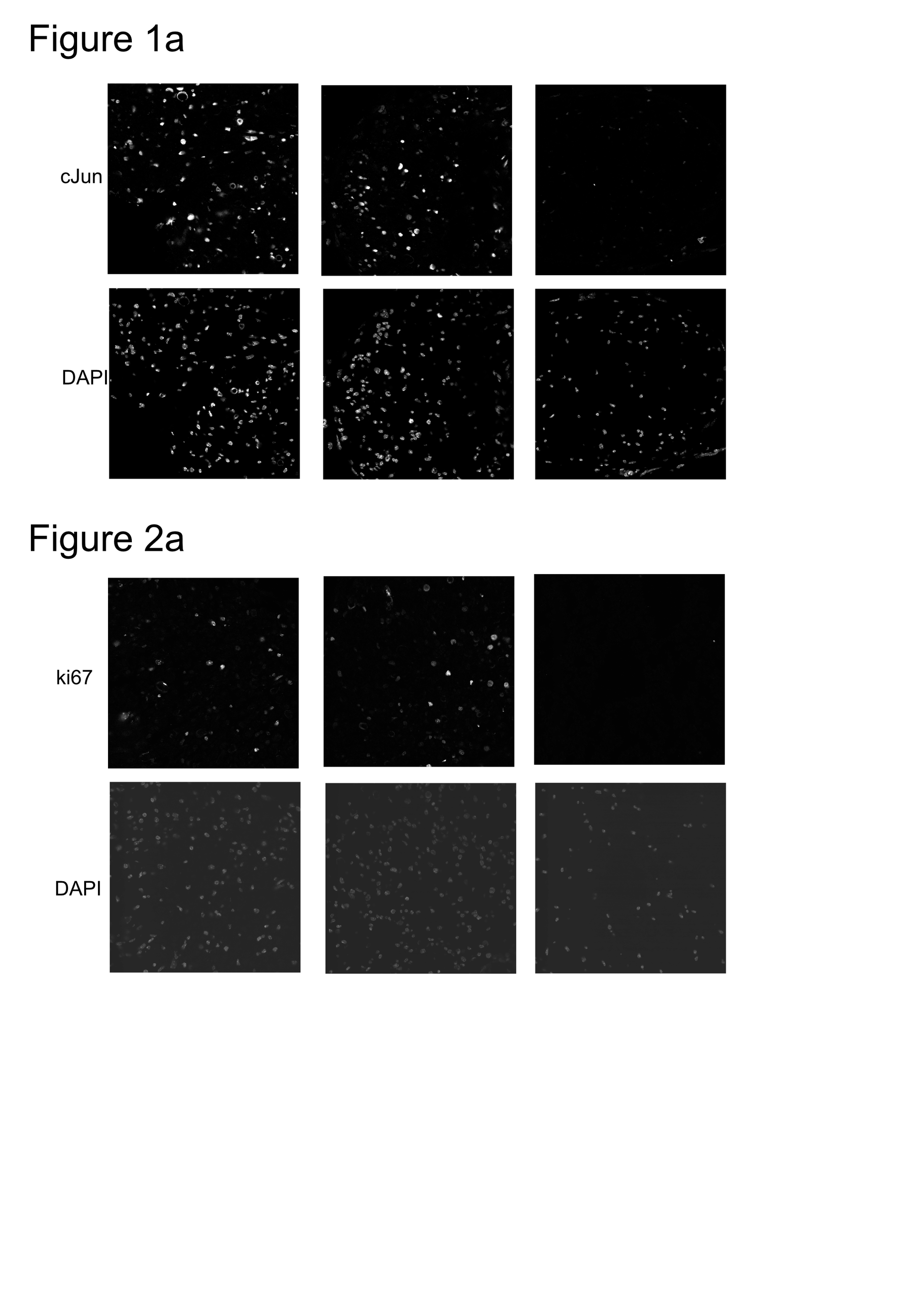

Supplement: S3 Fig — The separate channels for each image are shown without alterations of contrast or brightness and no cropping. (TIF) [file pone.0245944.s003.tif]

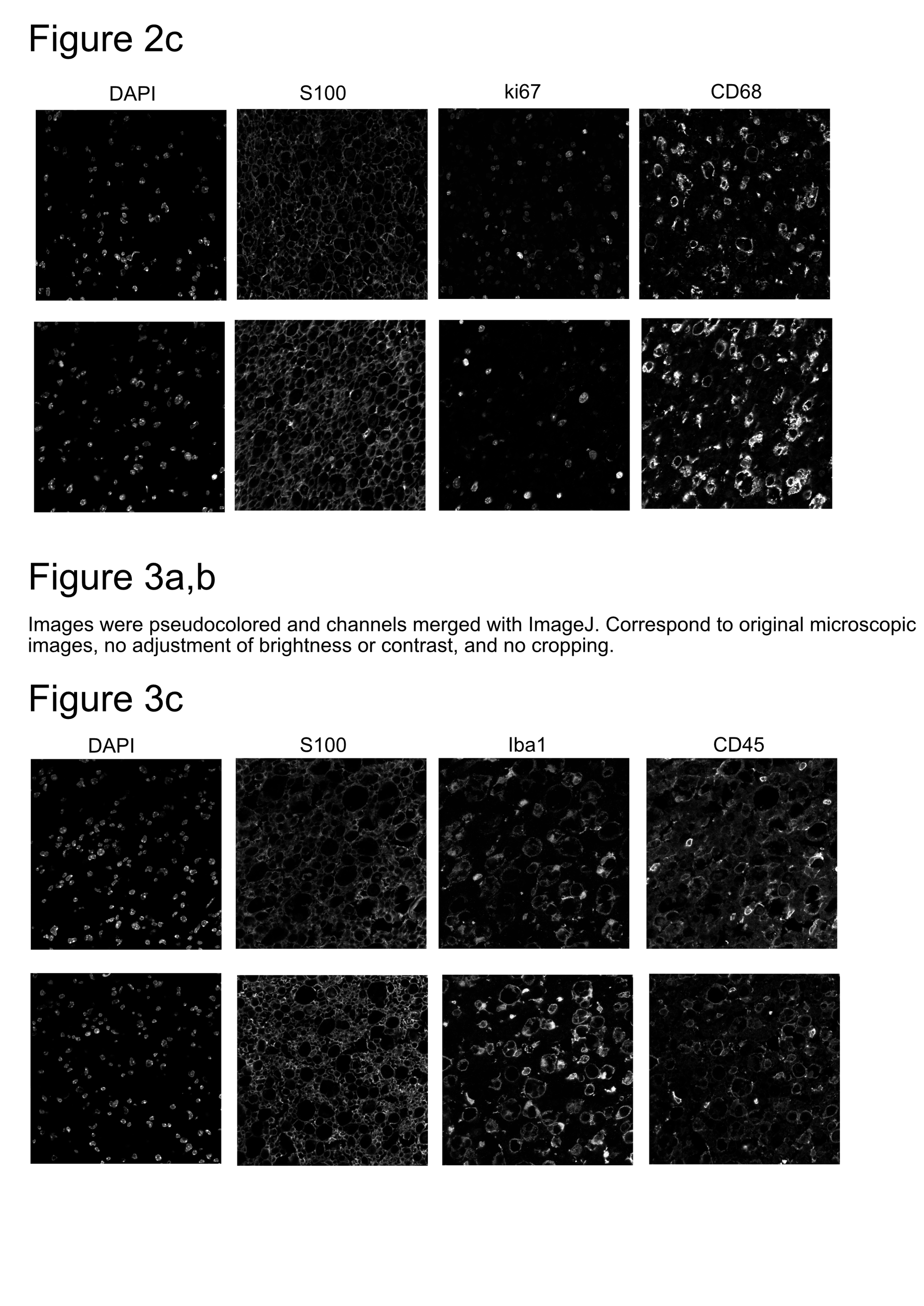

Supplement: S4 Fig — The separate channels for each image are shown without alterations of contrast or brightness and no cropping. (TIF) [file pone.0245944.s004.tif]
